# The association between skeletal muscle mass and functional capacity outcomes in Chinese older adults: a national community-based study

**DOI:** 10.3389/fpubh.2025.1645850

**Published:** 2025-10-20

**Authors:** Lin Chen, Xiaoqiang Lu, Zhongxin Zhu

**Affiliations:** ^1^Department of Rheumatology and Immunology, The First People's Hospital of Xiaoshan District, Xiaoshan Affiliated Hospital of Wenzhou Medical University, Hangzhou, China; ^2^Department of Scientific Research, The First People's Hospital of Xiaoshan District, Xiaoshan Affiliated Hospital of Wenzhou Medical University, Hangzhou, China; ^3^Department of Osteoporosis Care and Control, The First People's Hospital of Xiaoshan District, Xiaoshan Affiliated Hospital of Wenzhou Medical University, Hangzhou, China

**Keywords:** aging, sarcopenia, skeletal muscle mass, functional capacity, sex differences, CLHLS

## Abstract

**Background:**

Maintaining functional independence in older adults is a critical public health objective. Although skeletal muscle mass is recognized as a key contributor to functional capacity outcomes, the exact relationship between them among community-dwelling older adults requires further investigation.

**Methods:**

This cross-sectional study analyzed data from 13,322 participants aged ≥65 years from the 2018 Chinese Longitudinal Healthy Longevity Survey (CLHLS). Appendicular skeletal muscle mass index (ASMI) was calculated using a validated anthropometric equation, while functional capacity outcomes were assessed using basic and instrumental activities of daily living (BADL/IADL) scores. Multivariable linear regression, generalized additive models, and threshold effect analysis were employed to evaluate associations, with adjustments for demographic, socioeconomic, lifestyle, and comorbidity factors.

**Results:**

In fully adjusted models, ASMI showed significant inverse associations with both BADL score (*β* = −0.072, 95% CI: −0.103 to −0.042) and IADL score (*β* = −0.225, 95% CI: −0.290 to −0.159). Threshold effect analysis revealed sex-specific inflection points: below 6.2 kg/m^2^ in men and 5.2 kg/m^2^ below in women, ASMI was negatively associated with BADL/IADL scores, whereas above these thresholds, the associations weakened or reversed. Subgroup analyses indicated stronger effects among men, urban residents, and those with stroke or cardiovascular disease.

**Conclusion:**

Muscle mass demonstrates sex-specific, non-linear associations with functional capacity outcomes in older adults, identifying critical thresholds that may inform targeted strategies to preserve independence.

## Introduction

The global demographic transition toward population aging represents one of the most significant public health challenges of the 21st century, with China undergoing this transformation at an unprecedented scale and pace (1). Epidemiological data reveal a striking acceleration in aging dynamics: between 1990 and 2022, the proportion of China’s population aged ≥65 years increased 2.7-fold (from 5.57 to 14.9%), while the absolute number grew 3.3-fold (from 64 to 210 million individuals) (2). This demographic shift coincides with substantial gains in life expectancy (from 68.55 years in 1990 to 77.7 years in 2019), which is projected to reach 81.3 years by 2035 (3). Of particular concern are projections indicating that the disabled older adults population (≥65 years) will triple from 11.4 million in 2010 to 34.8 million by 2030, underscoring an urgent need for interventions that preserve functional autonomy to mitigate socioeconomic burdens (4).

Within this context, sarcopenia has emerged as a pivotal geriatric syndrome, characterized by the progressive, generalized loss of skeletal muscle mass and function (5). Contemporary understanding recognizes skeletal muscle not only as a contractile organ, but as a sophisticated endocrine platform that secretes myokines and exerkines capable of systemic metabolic regulation (6, 7). The musculoskeletal system’s dual roles in mechanical support and biochemical signaling make its age-related decline particularly consequential, with sarcopenia directly contributing to disability, frailty, and diminished quality of life (8, 9).

Although skeletal muscle mass is well-established as a contributor to physical capacity, growing evidence highlights the nuanced and complex nature of this relationship, particularly among older adults, where empirical studies have reported considerable inconsistencies (10, 11). For example, one systematic review concluded that the association between muscle mass and physical performance is weak or negligible (11). In contrast, a more recent comprehensive review demonstrated that reduced muscle mass is significantly associated with an increased risk of functional decline in older adults (10). Current clinical models predominantly assume linear relationships, potentially overlooking critical non-linear dynamics such as threshold effects and interactive mechanisms that could substantially improve intervention strategies. To better understand these complex associations, our study was designed to investigate the non-linear relationship between skeletal muscle mass and functional capacity in older adults.

## Methods

### Study design and population

We conducted a cross-sectional analysis using data from the 2018 wave of the Chinese Longitudinal Healthy Longevity Survey (CLHLS). Trained interviewers collected data through standardized face-to-face interviews. The study protocol was approved by the Biomedical Ethics Committee of Peking University, and all participants provided written informed consent. Publicly available data can be accessed through the CLHLS repository (https://opendata.pku.edu.cn/dataverse/pku/).

**Figure 1:**
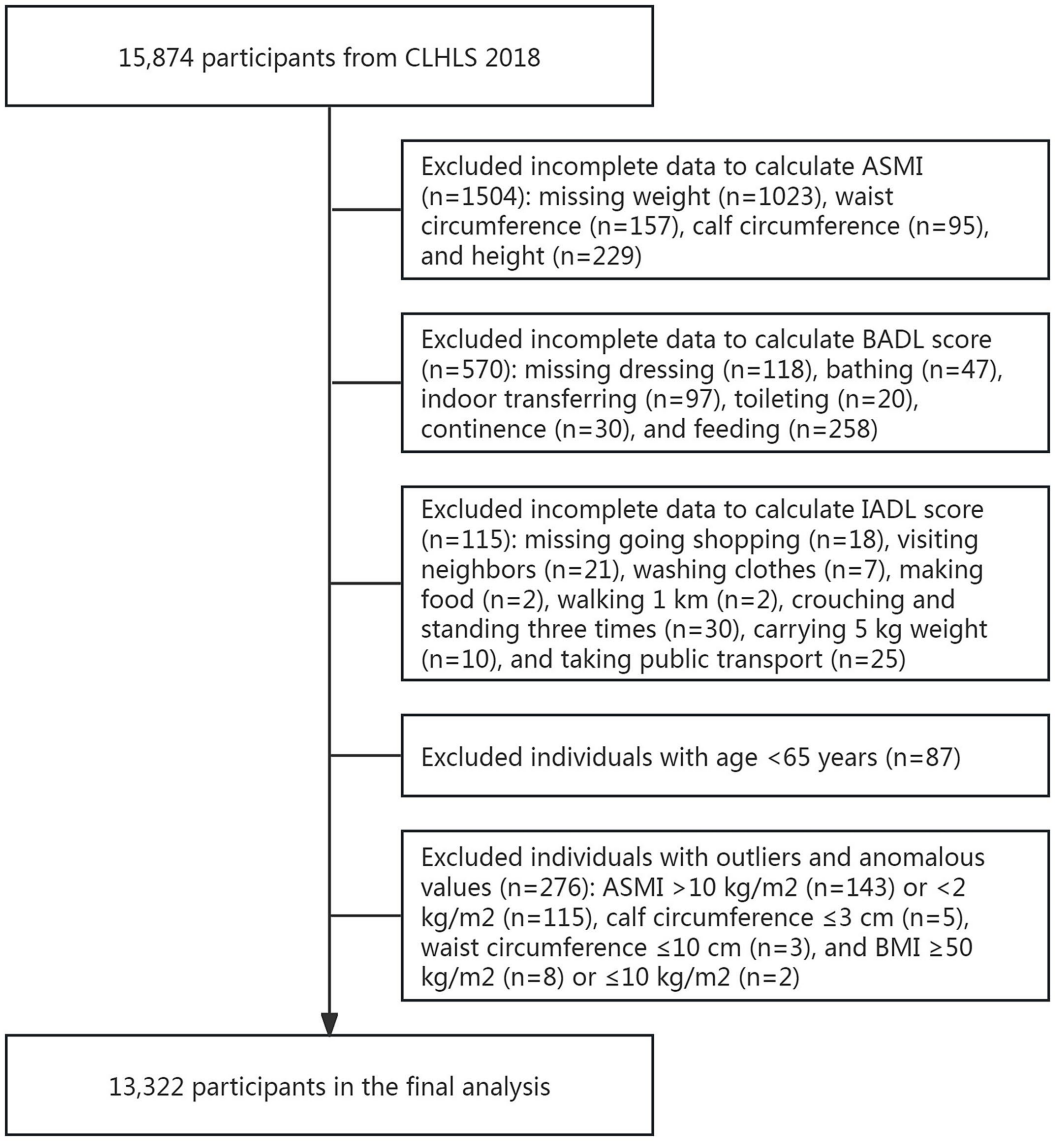
The flowchart of the participants selection.

From the initial sample (*n* = 15,874), we excluded participants with: (1) missing appendicular skeletal muscle index (ASMI, *n* = 1,504) measurements, (2) incomplete assessments of either basic activities of daily living (BADL, *n* = 570) or instrumental activities of daily living (IADL, *n* = 115) scores, (3) age < 65 years (*n* = 87); and outliers or anomalous values (*n* = 276). The final analytical sample comprised 13,322 participants (Figure 1).

### Variables

Appendicular skeletal muscle mass (ASM) was calculated using the validated Kawakami equation: ASM (kg) = 2.955 × sex (men = 1, women = 0) + 0.255 × weight (kg) - 0.130 × waist circumference (cm) + 0.308 × calf circumference (cm) + 0.081 × height (cm) - 11.897 (12). To account for body size, ASM was normalized by height squared to derive the ASMI (kg/m^2^).

Functional capacity outcomes were assessed using two validated scales. The BADL scale evaluated six domains: (1) dressing, (2) bathing, (3) indoor transferring, (4), toileting, (5) continence, and (6) feeding. The IADL scale assessed eight higher-level functional domains: (1) going shopping, (2) visiting neighbors, (3) washing clothes, (4) making food, (5) walking one kilometer, (6) crouching and standing three times, (7) carryng five kg weight, and (8) taking public transportation. In the present study, all items were scored on a 3-point scale (1 = fully independent to 3 = completely dependent), generating total scores ranging 6–18 for BADL and 8–24 for IADL, where higher scores indicated greater functional dependence.

The analysis adjusted for multiple potential confounders across four categories: (1) demographic factors including age (65–79, 80–94, and ≥95 years), sex, years of schooling (0, 1 to 6, or ≥ years), marital status (married, separated/divorced/widowed/never married), and residence (city, town, rural); (2) socioeconomic status measured by annual household income (<10,000; 10,000 to 30,000; 30,001 to 60,000; or >60,000 yuan); (3) lifestyle factors including status of exercise, smoke and drink; and (4) comorbidities including hypertension, diabetes, stroke or cardiovascular disease, cancer, and dementia.

### Statistical analyses

Participants were stratified into quartiles based on ASMI for baseline comparisons. Continuous variables were expressed as mean ± standard deviation, while categorical variables were presented as percentages. Between-group comparisons were performed using ANOVA for normally distributed continuous variables, Kruskal-Wallis tests for non-normally distributed continuous variables, and χ^2^ tests for categorical variables. The association between ASMI and functional capacity was examined using a series of progressively adjusted linear regression models: (1) an unadjusted model assessing crude associations; (2) a model adjusted for age and sex; and (3) a fully adjusted model incorporating demographic, socioeconomic, lifestyle, and comorbidity factors. To assess potential nonlinear associations, generalized additive models with smoothing splines were used for exploratory analysis. Where significant nonlinearity was indicated, piecewise linear regression was employed to identify and quantify threshold effects. Subgroup analyses were conducted to examine potential effect modifications by testing relevant interaction terms. All analyses were performed using R version 3.4.3 and EmpowerStats software. A two-tailed *p*-value < 0.05 was considered statistically significant.

## Results

### Study population characteristics

The characteristics of participants stratified by ASMI quartiles are presented in Table 1. Age exhibited a significant inverse trend, declining from 91.9 ± 10.1 years in Q1 to 79.3 ± 9.9 years in Q4. Sex distribution varied markedly, with men representing only 3.6% of Q1 but 83.8% of Q4. Higher ASMI was associated with greater educational level and elevated household income. Lifestyle behaviors also differed: regular exercise increased from 18.6% (Q1) to 42.3% (Q4), while smoking (Q1: 5.2%; Q4: 23.1%) and drinking (Q1: 6.7%; Q4: 24.3%) were more prevalent in higher quartiles. Comorbidities, including hypertension, diabetes, and stroke/cardiovascular disease, were more frequent in participants with higher ASMI. In contrast, dementia prevalence decreased from 3.2% (Q1) to 0.9% (Q4). Functional capacity outcomes showed poorer scores in both BADL (7.6 ± 2.8 in Q1 vs. 6.4 ± 1.5 in Q4) and IADL (16.7 ± 6.0 in Q1 vs. 11.0 ± 4.8 in Q4) among higher ASMI groups.

**Table 1:** Characteristics of participants based on appendicular skeletal muscle mass index quartile.

Appendicular skeletal muscle mass index	Q1	Q2	Q3	Q4	*p* value
Age (years)	91.9 ± 10.1	85.4 ± 10.9	82.3 ± 10.6	79.3 ± 9.9	<0.001
Sex (%)					<0.001
Men	3.6	28.8	64.6	83.8	
Women	96.4	71.2	35.4	16.2	
Years of schooling (%)					<0.001
0	68.9	46.5	30.2	20.0	
1 to 6	13.8	27.8	35.7	34.6	
≥ 7	3.4	11.2	19.3	31.1	
Unrecorded	14.0	14.5	14.8	14.4	
Marriage status (%)					<0.001
Married	15.3	35.0	51.2	63.4	
Separated/divorced/widowed/never married	83.9	64.0	47.7	35.8	
Unrecorded	0.8	1.0	1.0	0.8	
Residence (%)					<0.001
City	14.1	19.4	23.8	30.1	
Town	34.5	34.9	33.0	31.3	
Rural	51.4	45.7	43.2	38.7	
Annual household income (%)					<0.001
< 10,000 yuan	27.1	26.3	25.4	21.7	
10,000 to 30,000 yuan	27.1	24.7	22.5	22.3	
30,001to 60,000 yuan	17.2	16.7	17.7	18.0	
> 60,000 yuan	18.6	23.3	26.6	31.4	
Unrecorded	10.0	9.0	7.7	6.6	
Exercise (%)					<0.001
Yes	18.6	29.2	36.4	42.3	
No	79.7	69.7	62.4	56.4	
Not recorded	1.6	1.1	1.2	1.3	
Smoke (%)					<0.001
Yes	5.2	12.0	21.5	23.1	
No	93.9	86.6	77.7	76.1	
Not recorded	0.9	1.4	0.8	0.8	
Drink (%)					<0.001
Yes	6.7	10.2	17.2	24.3	
No	91.4	88.5	81.6	74.6	
Not recorded	1.9	1.3	1.2	1.2	
Hypertension (%)					<0.001
Yes	30.9	41.0	41.2	50.0	
No	60.8	52.2	52.6	45.3	
Not recorded	8.3	6.8	6.1	4.6	
Diabetes (%)					<0.001
Yes	4.4	8.7	10.3	13.4	
No	83.9	80.7	80.4	78.3	
Not recorded	11.6	10.6	9.3	8.3	
Stroke/cardiovascular disease (%)					<0.001
Yes	6.4	10.4	10.7	13.2	
No	82.0	79.6	79.5	78.5	
Not recorded	11.6	10.0	9.8	8.3	
Cancer (%)					<0.001
Yes	1.0	1.2	1.5	1.7	
No	81.4	83.5	83.4	85.0	
Not recorded	17.6	15.3	15.1	13.4	
Dementia (%)					<0.001
Yes	3.2	1.6	1.3	0.9	
No	84.9	87.7	88.7	89.9	
Not recorded	11.9	10.7	10.0	9.2	
Basic activity of daily living score	7.6 ± 2.8	6.9 ± 2.2	6.6 ± 1.8	6.4 ± 1.5	<0.001
Instrumental activities of daily living score	16.7 ± 6.0	13.5 ± 5.9	12.0 ± 5.3	11.0 ± 4.8	<0.001

### Associations between ASMI and BADL/IADL scores

**Figure 2:**
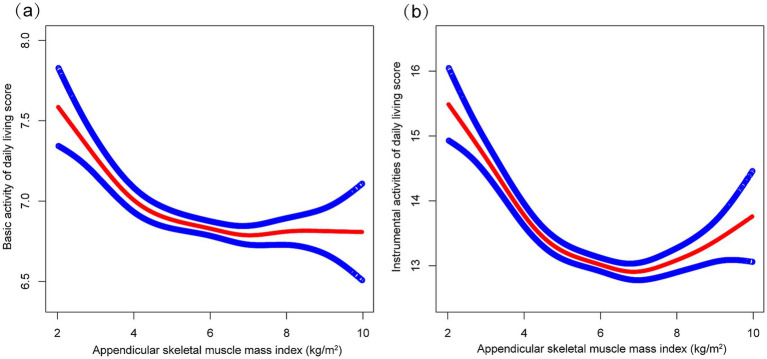
The correrations of appendicular skeletal muscle index with basic activities of daily living score **(a)** and instrumental activities of daily living score **(b)**. Higher scores indicate greater functional dependence. Age, sex, years of schooling, marriage status, residence, household income per year, status of exercise, smoke, drink, hypertension, diabetes, stroke/cardiovascular disease, cancer, and dementia were adjusted.

As shown in Table 2, multivariable regression analyses demonstrated significant inverse associations between ASMI and functional capacity outcomes, for both BADL (Model 3: *β* = −0.072, 95% CI: −0.103 to −0.042) and IADL scores (Model 3: *β* = −0.225, 95% CI: −0.290 to −0.159) across all models. Trend analyses confirmed these patterns, showing significant dose–response relationships, with Q3 demonstrating the strongest effects for both BADL and IADL scores in the fully adjusted model. These nonlinear relationships were further supported by the patterns illustrated in Figure 2.

**Table 2:** Associations of ASMI with BADL score and IADL score.

	Model 1^1^ β (95% CI)	Model 2^2^ β (95% CI)	Model 3^3^ β (95% CI)
BADL score	−0.288 (−0.311, −0.264) ^***^	−0.050 (−0.081, −0.020) ^**^	−0.072 (−0.103, −0.042) ^***^
ASMI quartile			
Q1	Reference	Reference	Reference
Q2	−0.661 (−0.764, −0.558)	−0.167 (−0.269, −0.065)	−0.175 (−0.273, −0.077)
Q3	−0.992 (−1.095, −0.889)	−0.225 (−0.342, −0.107)	−0.258 (−0.372, −0.145)
Q4	−1.152 (−1.254, −1.049)	−0.143 (−0.274, −0.013)	−0.218 (−0.346, −0.090)
P for trend	<0.001	0.042	0.001
IADL score	−1.367 (−1.427, −1.307) ^***^	−0.198 (−0.265, −0.131) ^***^	−0.225 (−0.290, −0.159) ^***^
ASMI quartile			
Q1	Reference	Reference	Reference
Q2	−3.145 (−3.411, −2.880)	−0.797 (−1.020, −0.574)	−0.796 (−1.009, −0.583)
Q3	−4.700 (−4.966, −4.435)	−1.002 (−1.258, −0.745)	−0.995 (−1.241, −0.748)
Q4	−5.614 (−5.880, −5.349)	−0.753 (−1.038, −0.468)	−0.812 (−1.089, −0.534)
P for trend	<0.001	<0.001	<0.001

^1^Model 1: Crude model.

^2^Model 2: Adjusted for age and sex.

^3^Model 3: Adjusted for age, sex, years of schooling, marriage status, residence, household income per year, status of exercise, smoke, drink, hypertension, diabetes, stroke/cardiovascular disease, cancer, and dementia.

^*^p < 0.05; ^**^p < 0.01; ^***^p < 0.001.

ASMI, appendicular skeletal muscle mass index; BADL, basic activity of daily living; IADL, instrumental activities of daily living.

### Subgroup analysis

**Figure 3:**
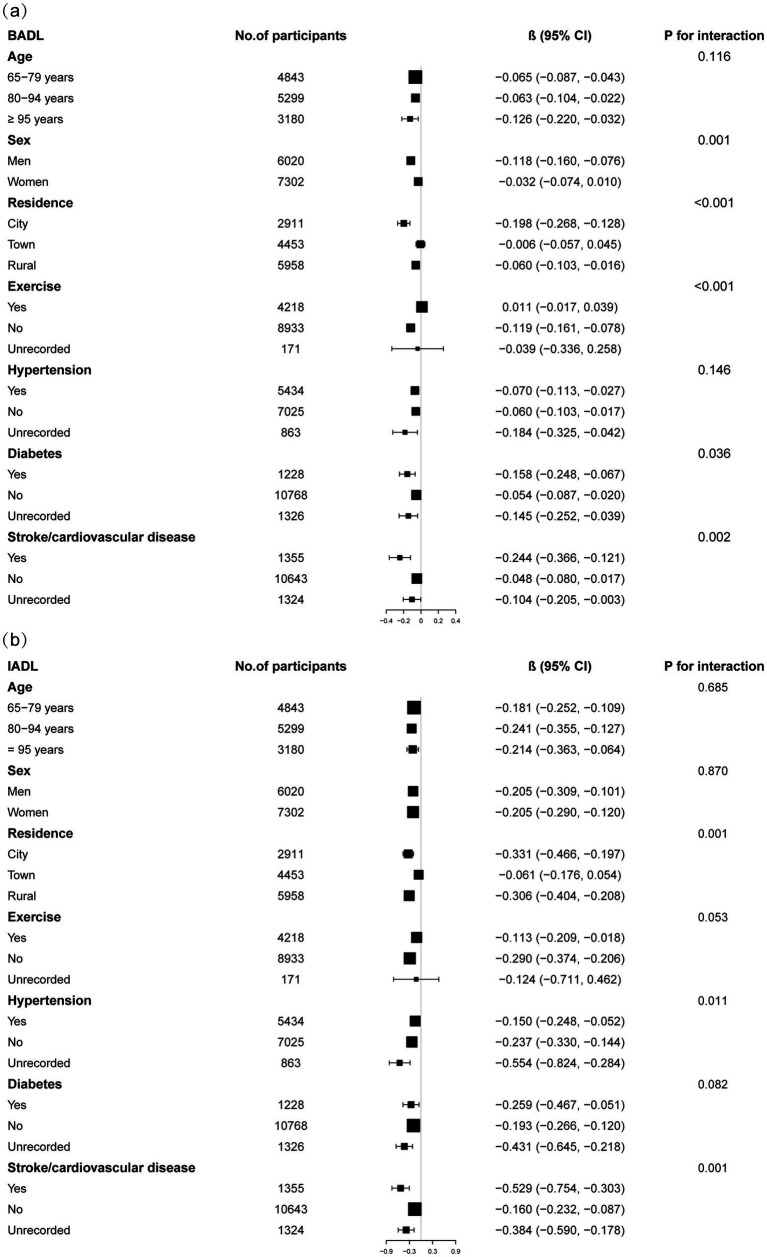
Subgroup analysis of the correrations of appendicular skeletal muscle index with basic activities of daily living score **(a)** and instrumental activities of daily living score **(b)**. Higher scores indicate greater functional dependence. Age, sex, years of schooling, marriage status, residence, household income per year, status of exercise, smoke, drink, hypertension, diabetes, stroke/cardiovascular disease, cancer, and dementia were adjusted. In the subgroup analysis, the model is not adjusted for the stratification variable itself.

**Figure 4:**
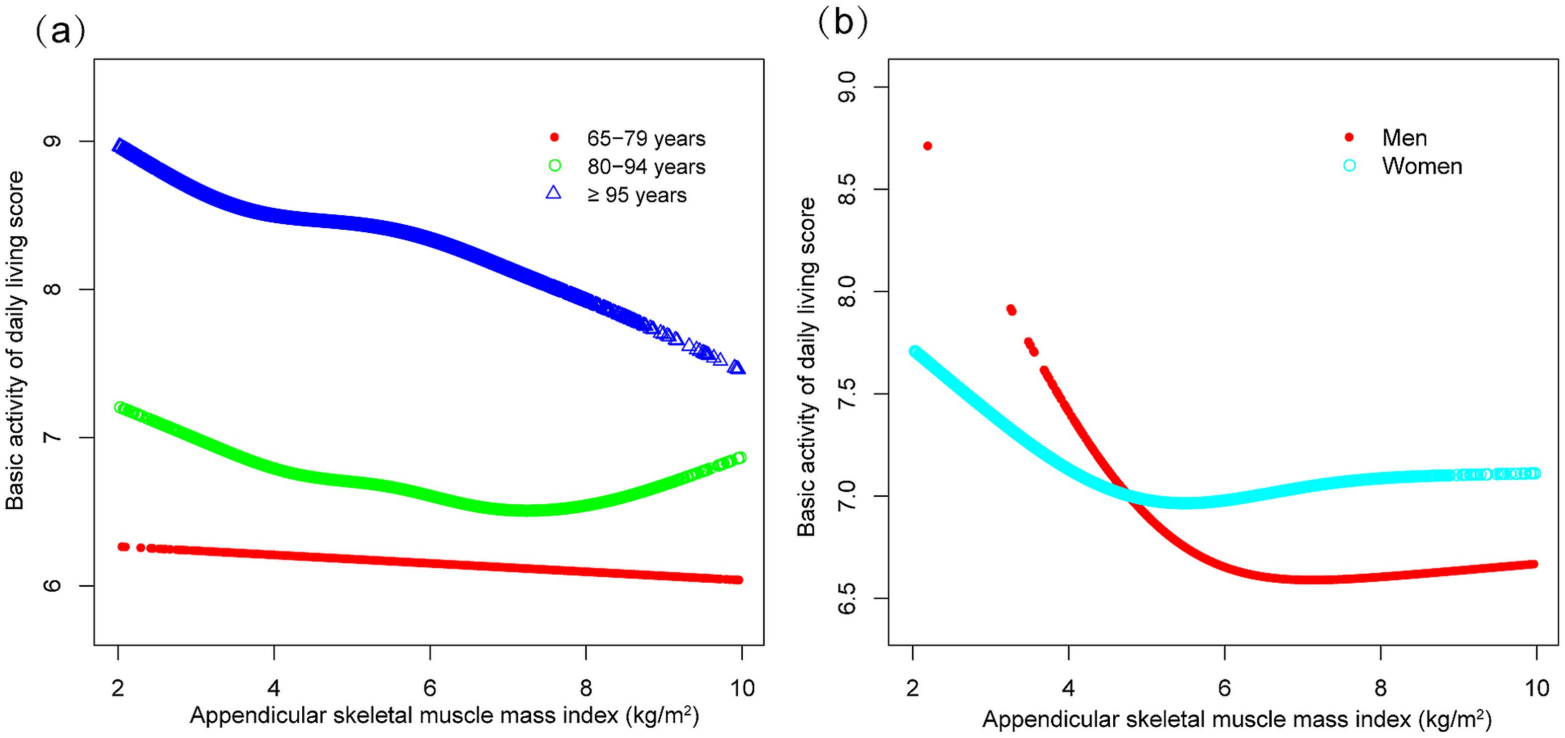
Curve associations between appendicular skeletal muscle index and basic activities of daily living score, stratified by age **(a)** and sex **(b)**. Higher scores indicate greater functional dependence. Age, sex, years of schooling, marriage status, residence, household income per year, status of exercise, smoke, drink, hypertension, diabetes, stroke/cardiovascular disease, cancer, and dementia were adjusted. In the subgroup analysis, the model is not adjusted for the stratification variable itself.

**Figure 5:**
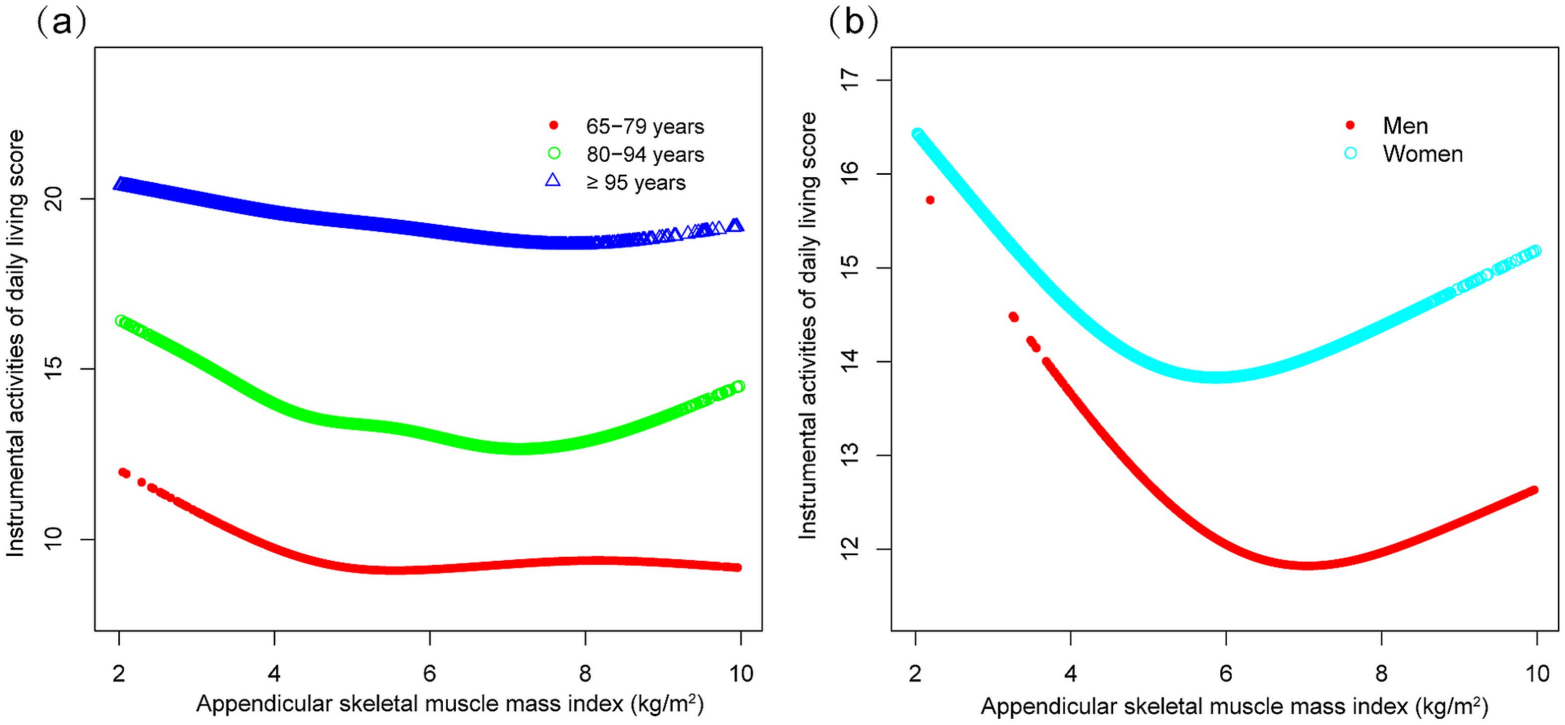
Curve associations between appendicular skeletal muscle index and instrumental activities of daily living score, stratified by age **(a)** and sex **(b)**. Higher scores indicate greater functional dependence. Age, sex, years of schooling, marriage status, residence, household income per year, status of exercise, smoke, drink, hypertension, diabetes, stroke/cardiovascular disease, cancer, and dementia were adjusted. In the subgroup analysis, the model is not adjusted for the stratification variable itself.

Subgroup analyses indicated differential associations between ASMI and functional decline (BADL/IADL) across demographic and clinical factors (Figure 3). For BADL (Figure 3a), significant interactions were observed for sex, residence, exercise, diabetes, and stroke/cardiovascular disease, with stronger negative effects in men (*β* = −0.118, 95% CI: −0.160 to −0.076), city residents (*β* = −0.198, 95% CI: −0.268 to −0.128), non-exercisers (*β* = −0.119, 95% CI: −0.161 to −0.078), diabetics (*β* = −0.158, 95% CI: −0.248 to −0.067), and stroke/cardiovascular patients (*β* = −0.244, 95% CI: −0.366 to −0.121). IADL (Figure 3b) showed significant interactions with residence, hypertension, and those with stroke/cardiovascular disease, with urban residents (*β* = −0.331, 95% CI: −0.466 to −0.197) and those with stroke/cardiovascular diseases (*β* = −0.529, 95% CI: −0.754 to −0.303) exhibiting steeper declines. Potential U-shaped nonlinear relationships stratified by age and sex are further illustrated in Figures 4, 5.

### Threshold effect analysis

Threshold effect analysis revealed significant sex-specific associations between ASMI and functional capacity measures (Table 3). Distinct inflection points were observed at 6.2 kg/m^2^ for men and 5.2 kg/m^2^ for women. In men, ASMI demonstrated strong inverse associations with both BADL (*β* = −0.400, 95% CI: −0.515 to −0.285) and IADL scores (*β* = −1.009, 95% CI: −1.293 to −0.724) below the 6.2 kg/m^2^ threshold. Above this threshold, these associations became non-significant (BADL: *β* = −0.018, 95% CI: −0.075 to 0.039; IADL: *β* = 0.081, 95% CI: −0.059 to 0.222). Women exhibited a comparable pattern below the 5.2 kg/m^2^ threshold, with negative associations for both BADL (*β* = −0.152, 95% CI: −0.226 to −0.079) and IADL (*β* = −0.692, 95% CI: −0.839 to −0.545). Above this threshold, the relationships became positive (BADL: *β* = 0.091, 95% CI: 0.016 to 0.165; IADL: *β* = 0.291, 95% CI: 0.143 to 0.439). The robustness of these threshold effects was confirmed by log-likelihood ratio tests (all *p* < 0.001).

**Table 3:** Threshold effect analysis of ASMI on BADL score and IADL score, stratified by sex.

	Adjusted *β* (95% CI), *p*-value
BADL score	
Men	
Inflection point	6.2
ASMI < 6.2	−0.400 (−0.515, −0.285), <0.001
ASMI > 6.2	−0.018 (−0.075, 0.039), 0.542
Log likelihood ratio	<0.001
Women	
Inflection point	5.2
ASMI < 5.2	−0.152 (−0.226, −0.079), <0.001
ASMI > 5.2	0.091 (0.016, 0.165), 0.017
Log likelihood ratio	<0.001
IADL score	
Men	
Inflection point	6.2
ASMI < 6.2	−1.009 (−1.293, −0.724), <0.001
ASMI > 6.2	0.081 (−0.059, 0.222), 0.256
Log likelihood ratio	<0.001
Women	
Inflection point	5.2
ASMI < 5.2	−0.692 (−0.839, −0.545), <0.001
ASMI > 5.2	0.291 (0.143, 0.439), <0.001
Log-likelihood ratio	<0.001

Age, years of schooling, marriage status, residence, household income per year, status of exercise, smoke, drink, hypertension, diabetes, stroke or cardiovascular disease, cancer, and dementia were adjusted.

ASMI, appendicular skeletal muscle mass index; BADL, basic activity of daily living; IADL, instrumental activities of daily living.

## Discussion

Our cross-sectional study reveals a non-linear association between ASMI and functional capacity in older adults, characterized by sex-specific inflection points at 6.2 kg/m^2^ for men and 5.2 kg/m^2^ for women. These findings challenge the prevailing linear paradigm of muscle mass-function relationships and support the development of threshold-based, sex-specific interventions to mitigate functional decline in aging populations.

Skeletal muscle mass is a well-established determinant of physical function in older adults, and its decline is linked to adverse health outcomes, including disability and reduced quality of life (13, 14). The loss of muscle mass reflects not only structural deterioration but also functional impairment, manifesting as diminished muscle strength and power—critical factors for maintaining independence (15, 16). However, inconsistencies persist in the literature, with some studies reporting no significant association between muscle mass and functional decline (17). These discrepancies may stem from methodological heterogeneity, including variations in muscle mass quantification, differences in body size normalization, and divergent definitions of functional outcomes.

The Asian Working Group for Sarcopenia recommends calf circumference as a practical screening tool for muscle mass loss in community settings (18). Although anthropometric proxies such as calf circumference and predictive algorithms provide convenient estimates, their accuracy in determining total-body skeletal muscle mass remains limited, prompting ongoing investigations into more sophisticated measurement techniques (19, 20). Our use of a validated equation that incorporates calf circumference as a key variable, aligns with previous studies in Asian populations (12, 21, 22). Notably, skeletal muscle mass plays a critical role in functional prognosis across diverse clinical settings, including acute stroke recovery (23), and demonstrates sex- and location-dependent associations with disability risk (24) —findings that corroborate our stratified analyses.

Our identified thresholds (6.2 kg/m^2^ for men and 5.2 kg/m^2^ for women) provide important context when compared with established sarcopenia diagnostic criteria. The Asian Working Group for Sarcopenia 2019 consensus recommends ASMI cut-offs of <7.0 kg/m^2^ for men and <5.4 kg/m^2^ for women (18), while the European Working Group on Sarcopenia suggests <7.0 kg/m^2^ for men and <5.5 kg/m^2^ for women (25). Our anthropometrically derived thresholds are notably lower, which may reflect methodological differences between anthropometric estimation and direct measurement techniques, as well as our focus on functional outcomes rather than sarcopenia diagnosis. Importantly, our thresholds represent inflection points for functional capacity rather than diagnostic cut-offs, suggesting that functional decline may begin at muscle mass levels below current diagnostic thresholds. For screening applications, the Kawakami equation used in this study provides a practical and cost-efficient tool for estimating muscle mass in community settings. In contrast to resource-intensive techniques such as dual-energy X-ray absorptiometry or bioelectrical impedance analysis, this approach utilizes readily obtainable anthropometric parameters, enabling scalable deployment in primary care, community health programs, and large-scale public health initiatives. For intervention strategies, older adults with muscle mass below the identified sex-specific thresholds may benefit from intensive, multimodal interventions incorporating progressive resistance training, dietary protein optimization, and task-specific functional exercises.

The age-related decline in skeletal muscle mass and function is driven by multifaceted biological mechanisms, including reductions in muscle fiber number and size, as well as qualitative alterations in muscle proteins (26). Myokines, which are secreted by muscle cells during contraction, play a pivotal role in regulating muscle metabolism and systemic physical function through autocrine and endocrine signaling (27). Concurrently, mitochondrial dysfunction in aging muscle contributes to deficits in exercise performance, gait stability, and insulin sensitivity, even among physically active older adults (28). Interventions such as combined strength training and tai chi have demonstrated efficacy in augmenting muscle mass in sarcopenic individuals (29), while high-protein dietary strategies can mitigate muscle loss during energy deficit (30). These findings underscore the potential for targeted lifestyle interventions to ameliorate functional decline.

Our study leverages a large sample and rigorous adjustment for confounders. However, several limitations warrant consideration. First, the cross-sectional design precludes causal inference, as the observed associations may reflect reverse causation or bidirectional relationships between muscle mass and functional capacity. Longitudinal studies with repeated measures are needed to clarify temporal sequences, establish causality, and address these concerns. Second, our reliance on anthropometric equations rather than direct measurement methods introduces potential measurement error and may lead to overestimation or underestimation of true muscle mass in individual cases. Therefore, the identified thresholds should be interpreted with considerable caution in clinical settings and require validation using gold-standard measurement techniques. Third, our subgroup analyses, while informative, were exploratory and not adjusted for multiple comparisons. These findings should therefore be interpreted as generating hypotheses for future validation rather than confirming definitive subgroup differences. Fourth, although the BADL/IADL scales are validated, they may not fully capture the multidimensional nature of functional decline in older adults. These scales primarily assess basic tasks and may miss important domains such as balance, gait speed, endurance, and cognitive-motor integration. Fifth, our decision not to independently adjust for BMI or waist circumference as covariates—guided by the validated Kawakami equation methodology—may introduce potential confounding by adiposity in the muscle mass–function relationship, particularly given the positive associations observed above the sex-specific thresholds.

## Conclusion

Our findings identify critical inflection points in the muscle mass-function relationship, suggesting that maintaining ASMI above sex-specific values (6.2 kg/m^2^ for men; 5.2 kg/m^2^ for women) is correlated with preserved autonomy in older adults. These observations highlight the potential value of moving beyond uniform recommendations toward precision strategies tailored to individual biomechanical and metabolic profiles. The results offer actionable insights for clinical practice and public health policy focused on supporting functional independence in aging populations.

## Data Availability

The datasets presented in this study can be found in online repositories. The names of the repository/repositories and accession number(s) can be found below: data are publicly accessible through the CLHLS open-access repository (https://opendata.pku.edu.cn/dataverse/pku/).

## References

[1] Gruber J Lin M Yang H Yi J. China's social health Insurance in the era of rapid population aging. JAMA Health Forum. (2025) 6:e251105. doi: 10.1001/jamahealthforum.2025.1105, PMID: 40279110

[2] Wang H Qin D Fang L Liu H Song P. Addressing healthy aging in China: practices and prospects. Biosci Trends. (2024) 18:212–8. doi: 10.5582/bst.2024.01180, PMID: 38987161

[3] Bai R Liu Y Zhang L Dong W Bai Z Zhou M. Projections of future life expectancy in China up to 2035: a modelling study. Lancet Public Health. (2023) 8:e915–22. doi: 10.1016/S2468-2667(22)00338-3, PMID: 37004714 PMC10188127

[4] Han Y Hu K Wu Y Fang Y. Future life expectancy with disability among elderly Chinese individuals: a forecast based on trends in stroke and dementia. Public Health. (2021) 198:62–8. doi: 10.1016/j.puhe.2021.06.013, PMID: 34364000

[5] Sayer AA Cooper R Arai H Cawthon PM Ntsama Essomba MJ Fielding RA. Sarcopenia. Nat Rev Dis Primers. (2024) 10:68. doi: 10.1038/s41572-024-00550-w, PMID: 39300120

[6] Saponaro F Bertolini A Baragatti R Galfo L Chiellini G Saba A. Myokines and microbiota: new perspectives in the endocrine muscle-gut Axis. Nutrients. (2024) 16:4032. doi: 10.3390/nu16234032, PMID: 39683426 PMC11643575

[7] Nunes EA Stokes T McKendry J Currier BS Phillips SM. Disuse-induced skeletal muscle atrophy in disease and nondisease states in humans: mechanisms, prevention, and recovery strategies. Am J Physiol Cell Physiol. (2022) 322:C1068–c1084. doi: 10.1152/ajpcell.00425.2021, PMID: 35476500

[8] Gauvain JB Mandigout S Pambet M Monseu M Gillain P Gautier J. Correlation between muscle mass and physical activity level in older adults at risk of falling: the FITNESS study. J Frailty Aging. (2024) 13:240–7. doi: 10.14283/jfa.2024.53, PMID: 39082768

[9] Petnehazy N Barnes HN Newman AB Kritchevsky SB Cummings SR Hepplen RT. Muscle mass, strength, power and physical performance and their association with quality of life in older adults, the study of muscle, mobility and aging (SOMMA). J Frailty Aging. (2024) 13:384–90. doi: 10.14283/jfa.2024.45, PMID: 39574257

[10] Visser M Sääksjärvi K Burchell GL Schaap LA. The association between muscle mass and change in physical functioning in older adults: a systematic review and meta-analysis of prospective studies. Eur Geriatr Med. (2025). doi: 10.1007/s41999-025-01230-y, PMID: 40407980 PMC12528211

[11] Riviati N Indra B. Relationship between muscle mass and muscle strength with physical performance in older adults: a systematic review. SAGE Open Med. (2023) 11:20503121231214650. doi: 10.1177/20503121231214650, PMID: 38033420 PMC10683395

[12] Kawakami R Miyachi M Tanisawa K Ito T Usui C Midorikawa T. Development and validation of a simple anthropometric equation to predict appendicular skeletal muscle mass. Clin Nutr. (2021) 40:5523–30. doi: 10.1016/j.clnu.2021.09.032, PMID: 34656948

[13] Distefano G Goodpaster BH. Effects of exercise and aging on skeletal muscle. Cold Spring Harb Perspect Med. (2018) 8:a029785. doi: 10.1101/cshperspect.a029785, PMID: 28432116 PMC5830901

[14] Hanna L Nguo K Furness K Porter J Huggins CE. Association between skeletal muscle mass and quality of life in adults with cancer: a systematic review and meta-analysis. J Cachexia Sarcopenia Muscle. (2022) 13:839–57. doi: 10.1002/jcsm.12928, PMID: 35156342 PMC8977976

[15] Reid KF Fielding RA. Skeletal muscle power: a critical determinant of physical functioning in older adults. Exerc Sport Sci Rev. (2012) 40:4–12. doi: 10.1097/JES.0b013e31823b5f13, PMID: 22016147 PMC3245773

[16] Mitchell WK Williams J Atherton P Larvin M Lund J Narici M. Sarcopenia, dynapenia, and the impact of advancing age on human skeletal muscle size and strength; a quantitative review. Front Physiol. (2012) 3:260. 22934016 10.3389/fphys.2012.00260 PMC3429036

[17] Schaap LA Koster A Visser M. Adiposity, muscle mass, and muscle strength in relation to functional decline in older persons. Epidemiol Rev. (2013) 35:51–65. doi: 10.1093/epirev/mxs006, PMID: 23221972

[18] Chen LK Woo J Assantachai P Auyeung TW Chou MY Iijima K. Asian working Group for Sarcopenia: 2019 consensus update on sarcopenia diagnosis and treatment. J Am Med Dir Assoc. (2020) 21:300–307.e2. doi: 10.1016/j.jamda.2019.12.012, PMID: 32033882

[19] Evans WJ Guralnik J Cawthon P Appleby J Landi F Clarke L. Sarcopenia: no consensus, no diagnostic criteria, and no approved indication-how did we get here? Geroscience. (2024) 46:183–90. doi: 10.1007/s11357-023-01016-9 37996722 PMC10828356

[20] McCarthy C Schoeller D Brown JC Gonzalez MC Varanoske AN Cataldi D. D(3) -creatine dilution for skeletal muscle mass measurement: historical development and current status. J Cachexia Sarcopenia Muscle. (2022) 13:2595–607. doi: 10.1002/jcsm.13083, PMID: 36059250 PMC9745476

[21] Ren L Tang Y Yang R Hu Y Wang J Li S. Plant-based dietary pattern and low muscle mass: a nation-wide cohort analysis of Chinese older adults. BMC Geriatr. (2023) 23:569. doi: 10.1186/s12877-023-04265-7, PMID: 37716958 PMC10505314

[22] Yang Y Wang Y Chen Q Li L Jia W. The association between low muscle mass and the risk of depressive symptoms: a cross-sectional study based on the Chinese longitudinal health longevity survey (CLHLS). Brain Behav. (2025) 15:e70267. doi: 10.1002/brb3.70267, PMID: 39910822 PMC11799061

[23] Honma K Honda Y Nagase M Nakao Y Harada T Sasanuma N. Impact of skeletal muscle mass on functional prognosis in acute stroke: a cohort study. J Clin Neurosci. (2023) 112:43–7. doi: 10.1016/j.jocn.2023.04.006, PMID: 37062242

[24] ZhouHDingXLuoM. The association between sarcopenia and functional disability in older adults. J Nutr Health Aging. (2024) 28:100016. doi: 10.1016/j.jnha.2023.100016, PMID: 38267154

[25] Cruz-Jentoft AJ Bahat G Bauer J Boirie Y Bruyère O Cederholm T. Sarcopenia: revised European consensus on definition and diagnosis. Age Ageing. (2019) 48:601. doi: 10.1093/ageing/afz046, PMID: 31081853 PMC6593317

[26] Larsson L Degens H Li M Salviati L Lee YI Thompson W. Sarcopenia: aging-related loss of muscle mass and function. Physiol Rev. (2019) 99:427–511. doi: 10.1152/physrev.00061.2017, PMID: 30427277 PMC6442923

[27] Lee JH Jun HS. Role of Myokines in regulating skeletal muscle mass and function. Front Physiol. (2019) 10:42. doi: 10.3389/fphys.2019.00042, PMID: 30761018 PMC6363662

[28] Grevendonk L Connell NJ McCrum C Fealy CE Bilet L Bruls YMH. Impact of aging and exercise on skeletal muscle mitochondrial capacity, energy metabolism, and physical function. Nat Commun. (2021) 12:4773. doi: 10.1038/s41467-021-24956-2, PMID: 34362885 PMC8346468

[29] Guo H Cao J He S Wei M Meng D Yu I. Quantifying the enhancement of Sarcopenic skeletal muscle preservation through a hybrid exercise program: randomized controlled trial. JMIR Aging. (2024) 7:e58175. doi: 10.2196/58175, PMID: 39621937 PMC11587998

[30] Pasiakos SM Margolis LM Orr JS. Optimized dietary strategies to protect skeletal muscle mass during periods of unavoidable energy deficit. FASEB J. (2015) 29:1136–42. doi: 10.1096/fj.14-266890, PMID: 25550460

